# The Effects of Oleic Acid and Palmitic Acid on Porcine Muscle Satellite Cells

**DOI:** 10.3390/foods13142200

**Published:** 2024-07-12

**Authors:** Shah Ahmed Belal, Jeongeun Lee, Jinryong Park, Darae Kang, Kwanseob Shim

**Affiliations:** 1 Department of Animal Biotechnology, Jeonbuk National University, Jeonju 54896, Republic of Korea; sabelal.sau@gmail.com (S.A.B.); drkang@jbnu.ac.kr (D.K.); 2 Department of Poultry Science, Sylhet Agricultural University, Sylhet 3100, Bangladesh; 3 Department of Agricultural Convergence Technology, Jeonbuk National University, Jeonju 54896, Republic of Korea; dlwjddms0625@naver.com; 4 Food Processing Research Group, Korea Food Research Institute, Wanju 55365, Republic of Korea; wlsfyd1321@naver.com

**Keywords:** proliferation, differentiation, myotube formation, triacylglycerol content, oleic acid, long-chain fatty acids

## Abstract

We aimed to determine the effects of oleic acid (OA) and palmitic acid (PA), alone or in combination, on proliferation, differentiation, triacylglycerol (TAG) content, and gene expression in porcine muscle satellite cells (PMSCs). Results revealed that OA-alone- and PA + OA-treated PMSCs showed significantly increased viability than those in the control or PA-alone-treated groups. No significant effects on apoptosis were observed in all three treatments, whereas necrosis was significantly lower in OA-alone- and PA + OA-treated groups than in the control and PA-alone-treated groups. Myotube formation significantly increased in OA-alone and PA + OA-treated PMSCs than in the control and PA-alone-treated PMSCs. mRNA expression of the myogenesis-related genes *MyoD1* and *MyoG* and of the adipogenesis-related genes *PPARα*, *C/EBPα*, *PLIN1*, *FABP4*, and *FAS* was significantly upregulated in OA-alone- and PA + OA-treated cells compared to control and PA-alone-treated cells, consistent with immunoblotting results for *MyoD1* and *MyoG*. Supplementation of unsaturated fatty acid (OA) with/without saturated fatty acid (PA) significantly stimulated TAG accumulation in treated cells compared to the control and PA-alone-treated PMSCs. These results indicate that OA (alone and with PA) promotes proliferation by inhibiting necrosis and promoting myotube formation and TAG accumulation, likely upregulating myogenesis- and adipogenesis-related gene expression by modulating the effects of PA in PMSCs.

## 1. Introduction

Intramuscular fat (IMF) is the most critical factor affecting the marbling and flavor of pork [1]. Lipids including triglycerides (triacylglycerol, TAG) are the key lipids in the skeletal muscle fat, and IMF contains phospholipids, which are responsible for pork flavor. To improve pork quality, methods to increase adipogenic differentiation must be identified. Fatty acids are essential nutrients that modulate adipogenic differentiation. Long-chain fatty acids (LCFAs) are natural compounds contributing to cellular metabolism in animal tissues [2]. A few influential factors, including saturation (saturated vs. unsaturated) and concentration (high vs. low) of LCFAs, accelerate myogenesis and adipocyte differentiation. Fatty acids may be specific agonists of the peroxisome proliferation-activated receptor (*PPAR*); specifically, *PPARγ* is essential for the adipose tissue differentiation process, and consequently, fatty acids play a significant role in modulating tissue differentiation by regulating *PPAR* activity and some transcription factors [3].

Skeletal muscle consists of about 40% of the body mass [4] and develops through myogenesis. Differentiation during myogenesis is tightly regulated by myotube formation [5]. Satellite cells are positioned in between the basal lamina and sarcolemma muscle fibers and have the potentiality of cell proliferation and differentiation [6]. After birth, skeletal muscle satellite cells play a significant role in skeletal muscle growth and regeneration [7]. For living organisms, free fatty acids (FFAs) are crucial to afford energy to cells by ATP synthesis through mitochondrial β-oxidation. Many investigations have reported that fatty acids modulate muscle function [8]. The most common FFAs are palmitic acid (PA; 16:0) and oleic acid (OA; 18:1).

PA (saturated fatty acid) is present naturally in vegetable oils [9]. It possesses a range of pharmacological activities, including antiviral, anti-inflammatory, analgesic, and some regulatory activities of lipid metabolism [10,11,12,13,14]. It promotes apoptosis by inducing cell cycle arrest in some human cells [15,16,17]. In addition, PA inhibits hepatoma cell proliferation by altering glucose metabolism [18]. Earlier studies established that low concentrations of PA induce the proliferation of Jurkat cells and lymphocytes, whereas high concentrations are cytotoxic [19,20,21]. Previous studies confirmed that OA exerts a proliferative effect on cultured muscle cells [22,23]. A few researchers have reported that PA is almost toxic to cells, whereas OA is cytoprotective or non-toxic to many types of cells [24,25,26,27]. PA may adversely affect skeletal muscle differentiation [3]. Moreover, FFAs may cause insulin resistance and the atrophy and myopathy of skeletal muscle [28]. The exact mechanisms behind the PA and OA effects on PMSCs have not yet been clearly elucidated. Several studies are available of PA and OA effects on the proliferation, necrosis, myogenesis, and adipogenic differentiation of satellite porcine cells. In this study, PMSCs were used to test the hypothesis that OA modulates the detrimental effects of PA on myogenesis, adipogenesis, and related mRNA expression. Therefore, we explored the protective effects of OA against PA on the proliferation, apoptosis, myogenic differentiation, and adipogenic differentiation of PMSCs.

## 2. Materials and Methods

### 2.1. Ethics Statement

The study protocol was certified by the Animal Ethics Committee of Jeonbuk National University (approval number: JBNU2020-0147). All experiments were conducted following the guidelines and regulations of Jeonbuk National University.

### 2.2. Preparation of PA and OA Samples

Two LCFAs, i.e., PA (C16:0) (Sigma Aldrich, Saint Louis, MO, USA, Catalog: P0500) and OA (C18:1) (Sigma Aldrich, Saint Louis, MO, USA, Catalog: P01383), were used in this experiment. Samples were prepared following the methods of Frago et al. [29]. Briefly, stock solutions of 200 mM concentrations were prepared in 100% ethanol (EtOH). Working solutions of 1 mM were made by incubating the fatty acids in media containing 10% endotoxin and fatty acid-free bovine serum albumin (BSA) at 37 °C for 30–60 min. This working solution was added to the cells to obtain the final fatty acid concentrations. All stock solutions were stored at −20 °C until further use.

### 2.3. PMSC Culture and Treatments

In this experiment, we used PMSCs isolated from skeletal muscle of 1-day-old male piglets following the procedure of [30]. Briefly, muscles were collected from femur skeletal muscle and washed with Dulbecco’s phosphate-buffered saline (Gibco, Carlsbad, CA, USA) supplemented with 1% penicillin–streptomycin (PS; Gibco). Subsequently, the chopped muscles were mixed with digestion solution (1 U/mL of dispase II (Roche, Indianapolis, IN, USA), 2 mg/mL of collagenase D (Roche), 0.25% trypsin-EDTA (Gibco), 10% PS in Dulbecco’s modified Eagle’s medium/nutrient mixture F-12 (DMEM/F12; Gibco, Carlsbad, CA, USA)) at 37 °C for 1 h. After digestion, the samples were filtered through a 100 µm and 40 µm cell strainer. The suspension was then centrifuged, the supernatant was removed, and cells were resuspended with 15% fetal bovine serum (FBS; Gibco) and 1% PS glutamine (PSG; Gibco) in DMEM/F12. After isolation, the PMSCs were cultured in DMEM/F12 (Gibco, Carlsbad, CA, USA) supplemented with 15% FBS (Gibco) and 1% penicillin–streptomycin–glutamine (Gibco, USA) in a humidified atmosphere of 5% CO_2_. As the cells reached 90% confluency, they were subcultured to increase the number of cells. After 24 h, the medium in the three treatment groups was replaced with solutions containing 20, 20, and 20 + 20 μM/mL concentrations of PA, OA, and PA + OA, respectively, and the cells therein were incubated for another 24 h. The cells in the control group were grown without fatty acid, and the medium was changed every 24 h.

### 2.4. Cell Viability Assay

Cell viability was measured using a cell-counting kit (CCK-8; CK04-11; Dojindo, Mashiki, Japan) after 24 h and 48 h of incubation. Briefly, cells (1 × 10^4^) were seeded in 96-well cell culture plates. Cells were then exposed to PA or OA (at 0, 10, 20, 50, 100, and 250 µM each) in a growth medium for different periods and then treated with CCK-8 solution following the manufacturer’s instruction. After 4 h of incubation at 37 °C, absorbance was measured using a microplate reader (Thermo Fisher Scientific, San Jose, CA, USA) at 450 nm. Cell viability was calculated using the following formula: cell viability = (OD_treated_ − OD_blank_)/(OD_control_ − OD_blank_) wells × 100, where OD represents optical density.

### 2.5. Giemsa Staining

Cells (3 × 10^5^) were seeded in a 6-well plate. After treatment with fatty acids, the medium was completely discarded, and the cells were fixed in methanol and air-dried for 5 min. Staining dye (Sigma, Burlington, MA, USA) was mixed with distilled water (1:20) and used for cell staining for 30 min. Subsequently, the cells were washed in tap water to remove excess stain and then dried. Finally, images were captured using an inverted microscope (CKX53, Olympus, Tokyo, Japan).

### 2.6. Flow Cytometry

For the flow cytometric analysis, PMSCs were cultured as controls and in PA-alone, OA-alone, and PA + OA treatments (at concentrations of 0, 20, 20, and 20 + 20 μM/mL, respectively) for 24 h and collected with 0.25% trypsin–ethylenediaminetetraacetate (EDTA). The preparation procedures for analyzing apoptosis and cell cycles followed a previous study [31]. All samples were analyzed by fluorescence-activated cell sorting (FACS) on a BD FACSCalibur flow cytometer using BD Cell Quest Pro 5.1 Software (Becton, Dickinson and Company, San Diego, CA, USA).

### 2.7. Apoptosis Assay Using Acridine Orange/Ethidium Bromide Staining Methods

Cells (3 × 10^5^) were seeded in a 6-well plate and cultured for 24 h; the medium in the three treatment groups was replaced with solutions containing 20, 20, and 20 + 20 μM/mL concentrations of PA, OA, and PA + OA, respectively, and the cells therein were incubated for 24 h. After removing the medium, the cells were fixed with a methanol–acetic acid mix (3:1) for 1 h at room temperature (RT; 24 °C). Subsequently, the cells were washed with ice-cold PBS after removing the methanol–acetic acid mix. The cell nuclei were counterstained with acridine orange/ethidium bromide (AO/EtBr) (100 µg/mL AO, 100 µg/mL EB) for 10 min and then analyzed under a fluorescence microscope with the ZEISS ZEN 3.7 imaging software (Carl Zeiss, Oberkochen, Germany).

### 2.8. DAPI Staining

PMSCs (2 × 10^5^ cells per well) were seeded in confocal dishes. After 24 h, the medium in the three treatment groups was replaced with solutions containing 20, 20, and 20 + 20 μM/mL concentrations of PA, OA, and PA + OA, respectively. The cells were fixed with 4% paraformaldehyde and then rinsed with PBS, after which they were permeabilized and blocked with a blocking solution (PBS containing 0.3% Triton X-100 and 3% BSA) for 1 h at RT (24 °C). Subsequently, the cells were washed thrice with 0.3% Triton X-100 in PBS and then incubated for 5 min at RT (24 °C) with 4′-6 diamidino-2-phenylindole (DAPI, 1:1000) to visualize the nuclei. Finally, images were captured using an LSM 880 confocal laser scanning microscope and analyzed with the ZEISS ZEN imaging software (Carl Zeiss, Oberkochen, Germany).

### 2.9. Wound-Healing Assay

The wound-healing activities of PMSCs were investigated following the methods described by Wang et al. [32]. Briefly, 80% confluent PMSCs were placed in 6-well plates with growth media for a 24 h incubation period, followed by treatment with different concentrations of PA, OA, or PA + OA for 24 h incubation period (*n* = 5 per group). The confluent cell layers were then scratched with a 10 µL pipette tip, washed with PBS, and treated with growth medium for another 24 h incubation period. Images were captured before and after the fatty acid treatment, and the percentage of migratory area was quantified using Adobe Photoshop PS7 software.

### 2.10. Oil Red O Staining

Oil red O staining was used to observe lipid accumulation in PMSCs. Approximately 3 × 10^5^ cells were seeded in each well of a 6-well plate in a growth medium, allowed to grow to 80–90% confluence, and then changed to adipogenic differentiation medium (DMEM (LG), 10% FBS, 1% PSG, IBMX (0.5 mM), dexamethasone (1 mM), indomethacine (100 µM), insulin (10 µg/mL)) to adipogenic differentiation. After the recommended incubation time, the cells in the three treatment groups were treated with 20, 20, and 20 + 20 μM/mL concentrations of PA, OA, and PA + OA, respectively, gently washed with PBS, and fixed at RT (24 °C) with 10% formalin. Subsequently, the cells were washed with distilled water after removing formalin and then incubated for 5 min at RT (24 °C) with 60% isopropanol. The isopropanol was discarded, and the cells were stained with Oil red O solution (0.5% Oil red O in isopropanol). Subsequently, the cells were washed with water after discarding the staining solution. The cells were then incubated with hematoxylin for 1 min, washed with water, and observed under a microscope (Olympus, Japan). Lipid content was measured by the direct extraction of Oil red O from the stained cells using isopropanol, and the absorbance at 492 nm was determined using a microplate reader (Multiskan, GO Microplate Spectrophotometer; Thermo Scientific, Waltham, MA, USA).

### 2.11. Analysis of Myotube Formation

PMSCs (2 × 10^5^ cells per well) were seeded in 6-well plates in a growth medium and allowed to grow up to 80–90% confluence and then changed to a myogenic differentiation medium (DMEM, with high glucose and 5% horse serum) to induce muscle differentiation. After 1 and 5 days, the multinucleated myotubes detected in a field (1 µm^2^ × 100) were counted by a phase-contrast microscope (PM 20; Olympus, Japan).

### 2.12. Measurement of Triacylglycerol Content

Cells cultured in 6-well plates were washed twice with ice-cold PBS, scraped off with 25 mM Tris–HCl (pH 7.5) containing 1.0 mM EDTA, and then homogenized with a microhomogenizer (Ieda Trading Corporation, Tokyo, Japan). Protein concentrations were measured using a detergent-compatible (DC) protein assay kit (Bio-Rad, Hercules, CA, USA). The triacylglycerol (TAG) content in the cell lysate was extracted using a chloroform–methanol mix (2:1 *v*/*v*) and quantified enzymatically using a triglyceride colorimetric assay kit (Cayman, Ann Arbor, MI, USA). The TAG content was normalized to the protein content in each well. All experiments were performed in triplicate and repeated at least thrice.

### 2.13. RNA Extraction and qRT-PCR

After treatment, total cellular RNA was extracted using TRIzol Reagent (Invitrogen, New York, NY, USA) following the manufacturer’s instructions. Total mRNA was quantified using a NanoDrop spectrophotometer (Thermo Fisher Scientific, San Jose, CA, USA). cDNA was synthesized using a cDNA synthesis kit (Bioneer, Daejeon, Republic of Korea). The reaction mix was prepared by adding the relevant primer pair, 1.0 µL of cDNA, and AMPIGENE^®^ qPCR green Mix (Enzo, San Diego, CA, USA) and made up to a total volume of 20 µL following the manufacturer’s protocol. Primer sequences of *Pax7, MyoD, MyoG, PPARγ*, CCAAT/enhancer-binding protein-α (*C/EBPα*), perilipin-1 (*PLIN1*), fatty acid binding protein-4 (*FABP4*), fatty acid synthase (*FAS*), lipoprotein lipase (*LPL*), and glyceraldehyde-3-phosphate dehydrogenase (*GAPDH*) genes are listed in Table S1 [31,33,34,35]. qPCR was performed in triplicate using a CFX96TM Real-Time PCR Detection System (Bio-Rad, USA). All data were normalized to *GAPDH* as a reference gene and calculated using the 2^−ΔΔ*C*T^ method [36].

### 2.14. Protein Extraction and Western Blot Analysis

Total protein was extracted from PMSCs using radioimmunoprecipitation assay buffer (Biosesang, Sungnum, Republic of Korea) containing a protease inhibitor (Thermo Fisher Scientific) after incubation on ice for 40 min. After centrifugation at 21,000 × *g* for 30 min, the supernatant was collected, and the protein concentration of the cell lysates was measured using the DC protein assay kit (Bio-Rad, USA). Proteins were subjected to sodium dodecyl sulfate–polyacrylamide gel electrophoresis using a 12% acrylamide gel and transferred onto polyvinylidene fluoride membranes. Membranes were blocked for 1.5 h at RT (24 °C) using 5% skimmed milk in Tris buffer solution (TBS) containing 0.5% Tween 20 (TBST), after which the membranes were rinsed with TBST and incubated overnight at 4 °C with the following primary antibodies: caspase 3 (1:1000; Novus Bio, Centennial, CO, USA), Bcl-2-associated X (1:1000; Santa Cruz Biotechnology, Dallas, TX, USA), Pax7 (1:1000; DSHB, Iowa City, IA, USA), MyoD (1:1000; Proteintech, Rosemont, IL, USA), MyoG (1:1000; Abcam, Cambridge, UK), GAPDH (1:5000; Invitrogen, Carlsbad, CA, USA). Membranes were rinsed with TBST and incubated for 1.5 h at (24 °C) with secondary antibodies. The corresponding horseradish peroxidase-conjugated secondary antibodies were used, namely goat anti-mouse IgG (1:2000; Thermo Fisher Scientific, San Jose, CA, USA) and anti-rabbit IgG (1:2000; Thermo Fisher Scientific, San Jose, CA, USA). After washing with TBST, immunoblots were visualized using an enhanced chemiluminescence kit (Thermo Fisher Scientific, San Jose, CA, USA), and images were captured using the iBright CL 100 Imaging system (Thermo Fisher Scientific, San Jose, CA, USA). All proteins were normalized with GAPDH.

### 2.15. Statistical Analysis

The effects of the treatments were analyzed by one-way analysis of variance using GraphPad Prism (Version 5). Tukey’s test was used for multiple comparisons. The statistical significance level was set at *p* < 0.05.

## 3. Results

### 3.1. Effects of PA and OA on Cell Proliferation

**Figure 1:**
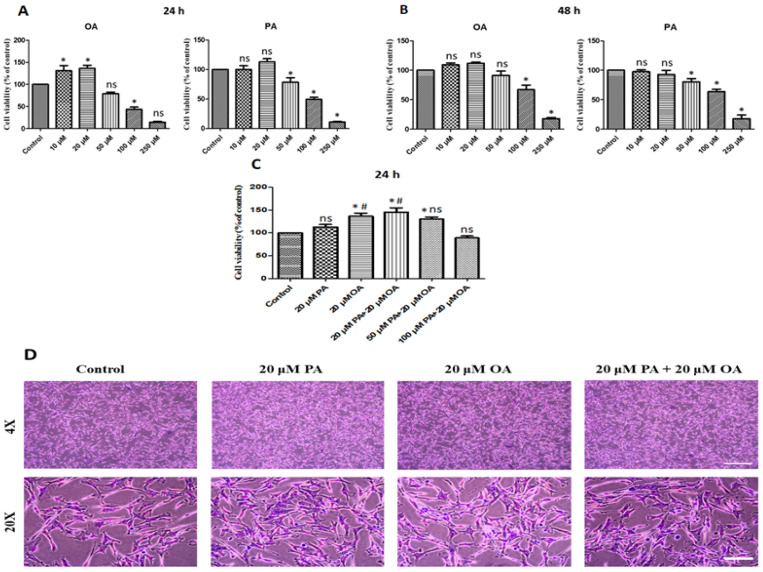
Effects of oleic acid (OA) and palmitic acid (PA) on cell viability at different physiological concentrations. (**A**,**B**) Porcine satellite cell viability: cells were incubated in separate experiments with 10 µM, 20 µM, 50 µM, 100 μM, and 250 μM OA and PA for 24 h and 48 h, respectively. (**C**) Porcine satellite cell viability: cells were incubated in separate experiments with 20 µM PA, 20 µM OA, 20 µM PA + 20 µM OA, 50 µM PA + 20 µM OA, and 100 µM PA + 20 µM OA for 24 h incubation. (**D**) Morphology visualized by Giemsa staining; scale bar = 100 and 20 μm for 4× and 20× of microscopic enhancement, respectively. Values are means ± SE, *n* = 5. * Indicates significance between treatment groups vs. control (*p* < 0.05), # Indicates significance between OA/PA + OA groups vs. PA group (*p* < 0.05), and ns indicates no significant difference. The control group consisted of porcine satellite cells that were untreated (no PA or OA).

Treatment of PMSCs with PA, OA, and PA + OA changed the cell viability after 24 and 48 h of incubation compared with the control group. After 24 h, increased and decreased cell proliferation were observed in PA-alone and OA-alone groups subjected to the dosages of 10–20 µM and 50–250 µM, respectively, compared with the control group (Figure 1A). After 48 h of incubation, there was no significant effect of 20 μM, whereas higher dosages decreased cell proliferation gradually (Figure 1B). Moreover, after 24 h of treatment, cell proliferation increased significantly (*p* < 0.05) in OA-alone- and PA + OA-treated groups subjected to 20 μM and 20 + 20 μM dosages, respectively, compared with that in the untreated control and PA-alone group subjected to the equivalent dosage of 20 μM (Figure 1C). These results indicate that treatment with a low concentration (20 µM) of PA and OA alone and 20 μM PA + 20 μM OA could markedly promote the proliferation of PMSCs rather than a high concentration. The promotion of cell proliferation was observed at low concentrations, but cytotoxicity was confirmed at high concentrations (particularly with PA). No significant morphological changes were observed in the control, PA, OA, or PA + OA groups (Figure 1D). Based on these results, we selected 20 µM as the optimal concentration of both PA and OA for the subsequent analyses.

### 3.2. Effects of PA and OA on the Cell Cycle

**Figure 2:**
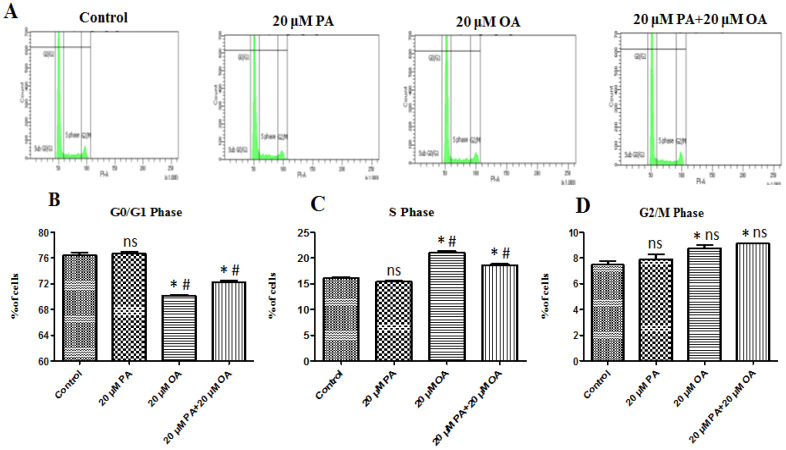
Effects of OA and PA on the cell cycle. (**A**) Cell cycle results with 20 µM PA, 20 µM OA, and 20 µM PA + 20 µM OA for a 24 h incubation period. (**B**–**D**) Changes in different cell cycle phases of PMSCs following PA and OA treatment. Values are means ± SE, *n* = 3. * Indicates significance between treatment groups vs. control (*p* < 0.05), # Indicates significance between OA/PA + OA groups vs. PA group (*p* < 0.05), and ns indicates no significant difference. The control group consisted of porcine satellite cells that were untreated (no PA or OA).

Cells treated with PA, OA, and PA + OA were analyzed to determine their cell cycle status (Figure 2A). In the control group, the percentage of cells in the G0/G1 phase was 76.43%, whereas it was 76.70, 70.17, and 72.23% in the PA-alone-, OA-alone-, and PA + OA-treated groups, respectively. In the OA-alone- and PA + OA-treated groups, the cell percentage significantly decreased in the G0/G1 phase and increased in the S phase (Figure 2B). No significant differences were observed in the G2/M phase (*p* > 0.05) (Figure 2D). These results indicate that OA (alone and in combination with PA) promotes cell proliferation by accelerating the transition from the G0/G1 phase to the S phase and are consistent with the results of the cell viability (Figure 2C).

### 3.3. Effects of PA and OA on Apoptosis and Necrosis

**Figure 3:**
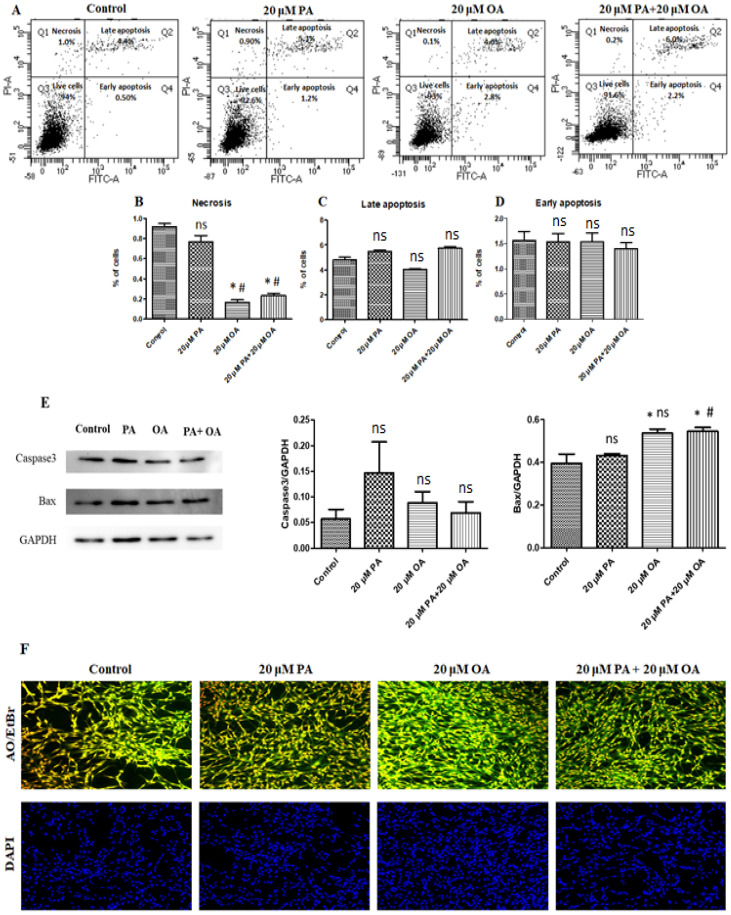
Analysis of cell necrosis, late apoptosis, and early apoptosis by FACS. (**A**) Porcine satellite cell apoptosis detection results with 20 µM PA, 20 µM OA, and 20 µM PA + 20 µM OA for a 24 h incubation period. (**B**) Proportion of necrotic cells. (**C**) Proportion of late apoptotic cells. (**D**) Proportion of early apoptotic cells. (**E**) Apoptosis-related protein expression. (**F**) Morphological observations using AO/EtBr staining and DAPI staining with confocal microscopy. Values are means ± SE, *n* = 3. * Indicates significance between treatment groups vs. control (*p* < 0.05), # Indicates significance between OA/PA + OA groups vs. PA group (*p* < 0.05), and ns indicates no significant difference. The control group consisted of porcine satellite cells that were untreated (no PA or OA).

The results of the apoptosis and necrosis assays are shown in Figure 3. Our results revealed that PA-alone-, OA-alone-, and PA + OA-treated groups did not show any significant differences in the extent of early and late apoptosis in PMSCs (Figure 3C,D). Necrosis was down-regulated by OA-alone- and PA + OA-treatment compared to control and PA-alone-treatment (*p* < 0.05) (Figure 3B). We also checked the cytoprotective effect of PA and OA on apoptosis-related proteins, like Caspase 3 and BAX (Figure 3E). The expression level of the apoptosis-inducing protein Caspase 3 was not significantly different among the groups (Figure 3E). However, expression levels of BAX significantly increased (*p* < 0.05) in the OA-alone- and PA + OA-treated groups compared to the control group (Figure 3E). Fluorescence microscopy was used to observe morphological changes after AO/EtBr and DAPI staining to evaluate cell viability and apoptosis (Figure 3F). Live cells showed green fluorescence, and those undergoing early apoptosis, late apoptosis, and necrosis showed yellowish-green and orange fluorescence, respectively. Live cells in OA-alone- and PA + OA-treated groups showed green fluorescence with normal nuclei. In contrast, the PA-alone-treated group subjected to a dosage of 20 µM presented more necrotic cells than the other groups. In DAPI staining, there were no significant morphological changes in the untreated control, PA, OA, or PA + OA groups (Figure 3F). Hence, treatment with OA alone or the PA + OA combination promoted cell growth and inhibited apoptosis.

### 3.4. Effects of PA and OA on Migration of PMSCs

**Figure 4:**
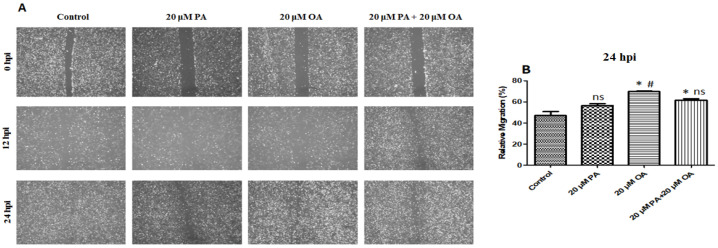
(**A**) Cell migration analysis performed by a wound healing assay; hpi indicates hour post incubation; scale bar = 100 μm. (**B**) Cell migratory area detection results with 20 µM PA, 20 µM OA, and 20 µM PA + 20 µM OA for a 24 h incubation period. Values are means ± SE, *n* = 5. * Indicates significance between treatment groups vs. control (*p* < 0.05), # Indicates significance between OA/PA + OA groups vs. PA group (*p* < 0.05), and ns indicates no significant difference. The control group consisted of porcine satellite cells that were untreated (no PA or OA).

Wound-healing assay results revealed that OA + PA co-treatment significantly reduced the migratory rate of PMSCs (Figure 4A,B). However, PA treatment did not affect the migratory capabilities of PMSCs compared with that in the control and PA + OA-treated groups.

### 3.5. Effects of PA and OA on Myotube Formation

**Figure 5:**
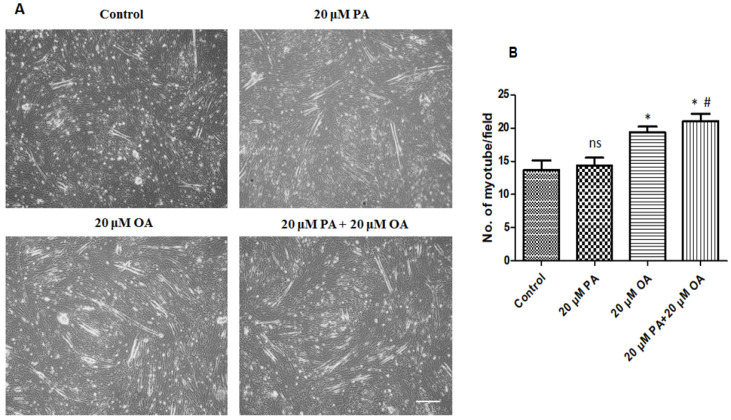
Effects of PA and OA on myogenic differentiation of PMSCs. Cells were cultured in growth media allow to 80–90% confluency and then switched to myogenic differentiation medium for a 120 h incubation period. (**A**) Cells were observed under a microscope; scale bar = 100 μm. (**B**) Number of myotubes. Values are means ± SE, *n* = 3. * Indicates significance between treatment groups vs. control (*p* < 0.05), # Indicates significance between OA/PA + OA groups vs. PA group (*p* < 0.05), and ns indicates no significant difference. The control group consisted of porcine satellite cells that were untreated (no PA or OA).

PMSCs grown in a growth medium (with 10% FBS) were switched to a differentiation medium (with 5% horse serum) to induce differentiation and were treated with PA and OA for 5 days to examine the effects of PA and OA on muscle differentiation. Myotubes with two or more nuclei were observed in PA-alone- and OA-alone-treated groups (Figure 5B). The number of myotubes in the OA-alone- and PA + OA-treated groups were significantly higher (*p* < 0.05) than that in the control and PA-alone-treated groups (Figure 5B).

### 3.6. Effects of PA and OA on the mRNA and Protein Expression of Myogenic Differentiation-Related Genes

**Figure 6:**
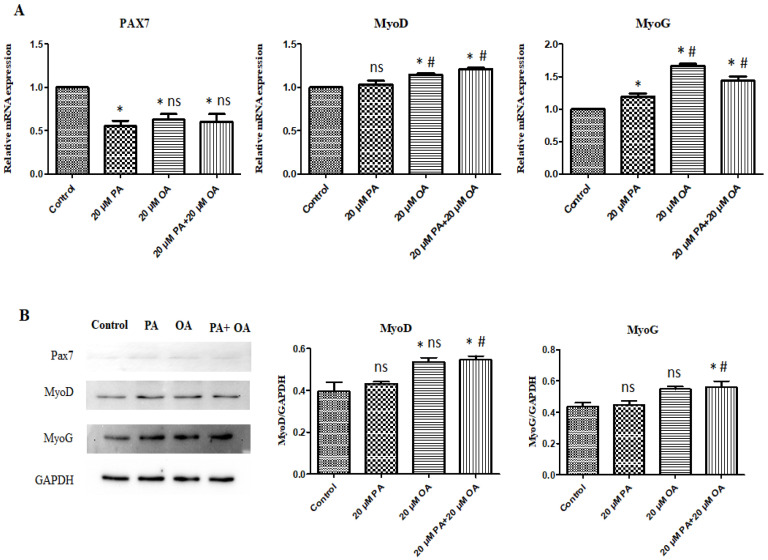
(**A**) Gene expression levels of myogenesis transcription factors under PA, OA, and PA + OA treatments. (**B**) Protein expression levels of myogenesis transcription factors under PA, OA, and PA + OA treatments. Values are means ± SE, *n* = 3. * Indicates significance between treatment groups vs. control (*p* < 0.05), # Indicates significance between OA/PA + OA groups vs. PA group (*p* < 0.05), and ns indicates no significant difference. The control group consisted of porcine satellite cells that were untreated (no PA or OA).

To determine the effects of PA and OA on the expression of myogenesis-related genes, we measured the mRNA levels of genes involved in the myogenic differentiation of PMSCs with PA, OA, or PA + OA. The results represented in Figure 6 indicate the effects of PA and OA (alone and in combination) on the expression of *Pax7*, *MyoD*, and *MyoG* genes at mRNA and protein levels using qPCR and Western blot analysis, respectively. Both OA and PA + OA treatment markedly affected the expression of myogenesis-related genes and proteins. Incubation of PMSC with 20 µM OA and 20 µM PA + 20 µM OA in the respective treatment groups resulted in a significant increase (*p* < 0.05) in the mRNA levels of *MyoD* and *MyoG* genes compared with those in the untreated control PMSCs and in PMSCs treated with 20 μM PA in the PA-alone treatment group (Figure 6A). In addition, the expression of *MyoD* and *MyoG* proteins was significantly (*p* < 0.05) upregulated by OA + PA co-treatment of PMSCs.

### 3.7. Effects of PA and OA on TAG Accumulation in PMSCs

**Figure 7:**
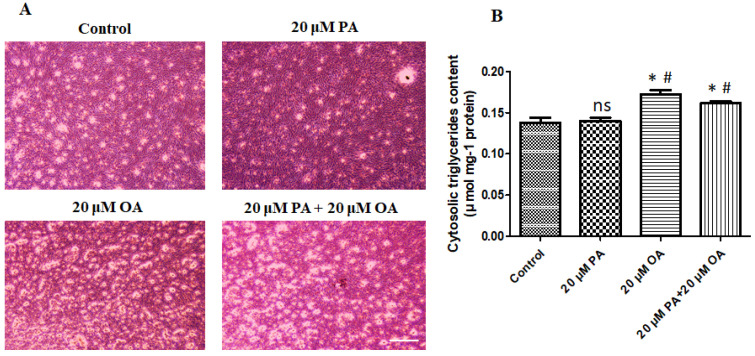
Effects of PA and OA on adipogenic differentiation of PMSCs. Cells were cultured in growth media to 80–90% confluency and then switched to adipogenic differentiation medium for a 120 h incubation period. (**A**) Oil red O staining indicating lipid droplets; scale bar = 100 μm. (**B**) Cytosolic TAG measured by enzymatic method during adipogenesis. Values are means ± SE, *n* = 5. * Indicates significance between treatment groups vs. control (*p* < 0.05), # Indicates significance between OA/PA + OA groups vs. PA group (*p* < 0.05), and ns indicates no significant difference. The control group consisted of porcine satellite cells that were untreated (no PA or OA).

As shown in Figure 7B, the supplementation of OA, both alone and with PA, significantly stimulated TAG accumulation in treated PMSCs (*p* < 0.05) compared with that in the control and PA-alone-treated PMSCs.

### 3.8. Effects of PA and OA on the mRNA Expression of Adipogenic Differentiation-Related Genes

**Figure 8:**
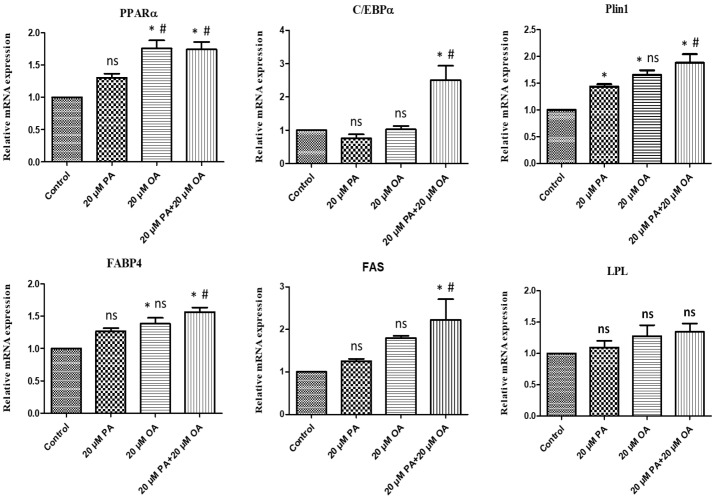
Gene expression levels of adipogenesis transcription factors under PA, OA, and PA + OA treatments. Values are means ± SE, *n* = 5. * Indicates significance between treatment groups vs. control (*p* < 0.05), # Indicates significance between OA/PA + OA groups vs. PA group (*p* < 0.05), and ns indicates no significant difference. The control group consisted of porcine satellite cells that were untreated (no PA or OA).

The mRNA expression of *PPARγ*, *C/EBPα*, *PLIN1*, *FABP4*, and *FAS* genes was upregulated in OA-alone- and OA + PA-treated PMSCs compared with that in the control and PA-treated PMSCs (Figure 8).

## 4. Discussion

In this study, we attempted to elucidate the effects of PA and OA on the proliferation, differentiation, and TAG accumulation in PMSCs. Fatty acids, particularly LCFAs, are sources of energy [37]. The PA and OA effects on cell viability were determined, and the results revealed that PMSC viability increased significantly after treatment with 20 µM of PA and OA each when incubated alone or together. Hardy et al. [17] reported that OA promoted cell proliferation via the phosphatidylinositol 3-kinase (PI3K) pathway. Further, OA increases the viability of C2C12 cells [8]. However, earlier studies showed that high concentrations of polyunsaturated fatty acids may inhibit cell proliferation in humans and rats [38,39,40]. In contrast, monounsaturated fatty acids, especially OA, are non-toxic and enhance cell proliferation in a few types of cell [24,25,26,27]. Double bonds are crucial for fatty acids to boost skeletal muscle cell proliferation. Hence, our results revealed that OA-containing double bonds might enhance the proliferation of PMSC. Lu et al. [41] showed that OA induces proliferation in rat cells. Furthermore, Mattern and Hardin [42] reported that OA treatment reduced the PA-induced apoptosis of vascular smooth muscle cells. Zeng et al. [43] confirmed that OA supplementation strongly alleviated pyroptosis caused by PA in HepG2 cells. Similarly, our study demonstrates that OA and PA co-treatment inhibited PA-induced apoptosis in PMSCs. These results suggested that cell proliferation may be enhanced by the supplementation of OA, both alone and with PA, in PMSCs. The wound-healing assay showed that OA + PA co-treatment significantly reduced the migratory rate of PMSCs, and PA treatment did not affect the migratory capabilities of PMSCs. However, PA treatment at concentrations of 12.5–50 μM significantly reduced the migratory area of porcine vascular endothelial cells [44].

Skeletal muscle formation, or myogenesis, is a complex process involving myoblast proliferation, followed by morphological, biochemical, and molecular modifications, resulting in the formation of multinucleated myotubes [45]. The formation of myotubes was increased (*p* < 0.05) in the OA-alone- and PA + OA-treated groups compared with that in the control and PA-treated groups. Zhang et al. [46] reported that pretreatment with PA inhibited skeletal muscle differentiation and decreased myotube formation in C2C12 skeletal muscle cells. Studies on the expression of myogenesis-related genes in PMSCs exposed to PA and PA + OA are limited. OA-alone- and PA + OA-treated PMSCs showed a significantly upregulated expression of *Pax7* and *MyoD* genes compared with that in PA-alone-treated PMSCs. *MyoD* and *MyoG* are key factors in myogenesis. Upregulation of *Pax7* expression increases the expression of these two myogenic regulatory factors [47]. In skeletal muscles, *MyoD* is the only factor expressed [48]. It regulates muscle cell differentiation by regulating the cell cycle and is required for myogenic initiation. *MyoD* expression reflects the activation and differentiation of bovine satellite cells (BSCs) [49] and plays a vital role in the differentiation of precursor cells into myogenic cells [50]. *MyoG* expression was significantly upregulated in OA-alone- and PA + OA-treated groups than in the untreated control and PA-alone- treated groups. The *MyoG* gene encodes a specific transcription factor that induces the late stage of myogenesis and is essential for skeletal muscle formation [51,52]. Li et al., [53] showed that OA accelerates *MyoG* expression in BSCs, which participate in myoblast fusion to form myotubes [54]. The increased expression of *MyoD* and *MyoG* suggests that muscle fiber development is accelerated, and their downregulated expression indicates that muscle development is inhibited.

OA (both alone and when co-supplemented with PA) significantly increased TAG accumulation in the treated PMSCs compared with that in the control and PA-alone-treated PMSCs. Earlier studies have demonstrated that OA is one of the most abundant fatty acids among liver triglycerides and induces lipid droplet formation in HepG2 cells and primary cultured human hepatocytes [55,56]. In primary cultured BSCs, unsaturated (OA) fatty acids significantly stimulate TAG accumulation compared with that obtained using saturated (PA) fatty acids [57]. Another study revealed that the induction of palmitoleic acid differentiates BSCs by promoting TAG accumulation [58]. The *PAT* family protein *PLIN* is a critical marker for analyzing cellular lipid accumulation and metabolism. *PLIN1* and *PLIN2* are activated or inhibited by the co-activator of *PPARγ*. This study indicates that OA-alone- and PA + OA-treated PMSCs exhibit a significant upregulation of *PLIN1* expression related to lipid droplet formation after differentiation.

Adipogenic differentiation is a complex process involving de novo fatty acid synthesis attributed to *FAS* and transcriptional control attributed to *PPARγ* and *C/EBPα*. The mRNA expression of *PPARγ*, *C/EBPα*, *PLIN1*, *FABP4*, and *FAS* genes was upregulated by OA supplementation (alone and in combination with PA), and both treatments must have stimulated TAG accumulation. The *PPAR* signaling pathway is principally involved in regulating cell proliferation, differentiation, and lipid metabolism [59]. Upregulated expression of *LPL* and *FABP4* expression promotes fatty acid uptake and esterification [53]. The upregulated FABP4 and *PPARγ* expression confirmed the adipogenic differentiation ability [33]. OA stimulated the differentiation of porcine cells by upregulating *PPARγ* and *C/EBPα* gene expression, whereas PA and other saturated fatty acids had no effects [60]. In another study, Sanosaka et al. [61] reported that PPARγ was induced by OA treatment in porcine intramuscular adipocytes. These results are consistent with those reported in this study and indicate that OA has the potential to regulate gene expressions and muscle differentiation against PA activity. Other fatty acids, e.g., myristic acid, increased the mRNA expression of *PPARγ*, *LPL*, and *FAS* genes [1] and palmitoleic acid upregulated the expression of *PPARγ* and *C/EBPα* genes [58]. Finally, OA and co-treatment with PA played a significant role in proliferation, apoptosis, myogenesis, and adipogenesis, suggesting that proper amounts of PA and OA in the diets could impact meat quality. These results may be helpful for further studies using models of myogenesis and adipogenesis, and for studies on cultured meat.

## 5. Conclusions

In this study, we describe evidence that OA, both alone and in combination with PA, promotes cell proliferation. Changes in the cell cycle from G0/G1 phase to S phase demonstrate that PA and OA accelerate the proliferation of PMSCs. Oleic acid alone and co-treatment with PA upregulated expressions of the myogenesis-related genes *MyoD* and *MyoG*. In addition, the mRNA expression of *PPARα*, *C/EBP4*, *PLIN1*, *FABP4*, and *FAS* genes was upregulated by OA (both alone and with PA), implicated in adipogenic differentiation and TAG accumulation. Therefore, this study suggests that OA impacts myogenesis and adipogenesis in PMSCs by modulating the effects of PA. However, more studied are required to find out the precise contribution of OA and PA in myogenesis. In addition, the regulatory factors and mechanisms determining myogenic and adipogenic differentiation of PMSCs need further exploration.

## Data Availability

The original contributions presented in the study are included in the article; further inquiries can be directed to the corresponding author.

## Supplementary Materials

The following supporting information can be downloaded at: https://www.mdpi.com/article/10.3390/foods13142200/s1, Table S1: Summary of primers of target reference genes used for qPCR analysis.

## References

[1] Lu N.S., Shu G., Xie Q.P., Zhu X.T., Gao P., Zhou G.X., Wang S., Wang L.N., Xi Q.Y., Zhang Y.L. (2014). Myristic Acid (MA) Promotes Adipogenic Gene Expression and the Differentiation of Porcine Intramuscular Adipocyte Precursor Cells. J. Integr. Agric..

[2] Schonfeld P., Wojtczak L. (2016). Short- and medium-chain fatty acids in energy metabolism: The cellular perspective. J. Lipid Res..

[3] Hurley M.S., Flux C., Salter A.M., Brameld J.M. (2006). Effects of fatty acids on skeletal muscle cell differentiation in vitro. Br. J. Nutr..

[4] Huard J., Li Y., Fu F.H. (2002). Muscle injuries and repair: Current trends in research. J. Bone Jt. Surg..

[5] Chal J., Pourquié O. (2017). Making muscle: Skeletal myogenesis in vivo and in vitro. Development.

[6] Wang H., He K., Zeng X., Zhou X., Yan F., Yang S., Zhao A. (2021). Isolation and identification of goose skeletal muscle satellite cells and preliminary study on the function of C1q and tumor necrosis factor-related protein 3 gene. Anim. Biosci..

[7] Rhoads R.P., Fernyhough M.E., Liu X., McFarland D.C., Velleman S.G., Hausman G.J., Dodson M.V. (2009). Extrinsic regulation of domestic animal-derived myogenic satellite cells II. Domest. Anim. Endocrinol..

[8] Lee J.H., Tachibana H., Morinaga Y., Fujimura Y., Yamada K. (2009). Modulation of proliferation and differentiation of C2C12 skeletal muscle cells by fatty acids. Life Sci..

[9] Mancini A., Imperlini E., Nigro E., Montagnese C., Daniele A., Orrù S., Buono P. (2015). Biological and nutritional properties of palm oil and palmitic acid: Effects on health. Molecules.

[10] Librán-Pérez M., Pereiro P., Figueras A., Novoa B. (2019). Antiviral activity of palmitic acid via autophagic flux inhibition in zebrafish (*Danio rerio*). Fish. Shellfish Immunol..

[11] Mayneris-Perxachs J., Guerendiain M., Castellote A.I., Estruch R., Covas M.I., Fitó M., Salas-Salvadó J., Martínez-González M.A., Aros F., Lamuela-Raventós R.M. (2014). Plasma fatty acid composition, estimated desaturase activities, and their relation with the metabolic syndrome in a population at high risk of cardiovascular disease. Clin. Nutr..

[12] Sawada K., Kawabata K., Yamashita T., Kawasaki K., Yamamoto N., Ashida H. (2012). Ameliorative effects of polyunsaturated fatty acids against palmitic acid-induced insulin resistance in L6 skeletal muscle cells. Lipids Health Dis..

[13] Moon M.L., Joesting J.J., Lawson M.A., Chiu G.S., Blevins N.A., Kwakwa K.A., Freund G.G. (2014). The saturated fatty acid, palmitic acid, induces anxiety-like behavior in mice. Metabolism.

[14] Zhou B., Zhang J., Zhang Q., Permatasari F., Xu Y., Wu D., Yin Z., Luo D. (2013). Palmitic acid induces production of proinflammatory cytokines interleukin-6, interleukin-1β, and tumor necrosis factor-α via a NF-κB-dependent mechanism in HaCaT keratinocytes. Mediat. Inflamm..

[15] Hsiao Y.H., Lin C.I., Liao H., Chen Y.H., Lin S.H. (2014). Palmitic acid-induced neuron cell cycle G2/M arrest and endoplasmic reticular stress through protein palmitoylation in SH SY5Y human neuroblastoma cells. Int. J. Mol. Sci..

[16] Pereira D.M., Correia-da-Silva G., Valentão P., Teixeira N., Andrade P.B. (2014). Palmitic acid and ergosta-7, 22-dien-3-ol contribute to the apoptotic effect and cell cycle arrest of an extract from *Marthasterias glacialis* L. in neuroblastoma cells. Mar. Drugs.

[17] Hardy S., Langelier Y., Prentki M. (2000). Oleate activates phosphatidylinositol 3-kinase and promotes proliferation and reduces apoptosis of MDA-MB-231 breast cancer cells, whereas palmitate has opposite effects. Cancer Res..

[18] Lin L., Ding Y., Wang Y., Wang Z., Yin X., Yan G., Zhang L., Yang P., Shen H. (2017). Functional lipidomics: Palmitic acid impairs hepatocellular carcinoma development by modulating membrane fluidity and glucose metabolism. Hepatology.

[19] Takahashi H.K., Cambiaghi T.D., Luchessi A.D., Hirabara S.M., Vinolo M.A., Newsholme P., Curi R. (2012). Activation of survival and apoptotic signaling pathways in lymphocytes exposed to palmitic acid. J. Cell Physiol..

[20] Gorjao R., Cury-Boaventura M.F., de Lima T.M., Curi R. (2007). Regulation of human lymphocyte proliferation by fatty acids. Cell Biochem. Funct..

[21] Lima T.M., Kanunfre C.C., Pompeia C., Verlengia R., Curi R. (2002). Ranking the toxicity of fatty acids on Jurkat and Raji cells by flow cytometric analysis. Toxicol. In Vitro.

[22] Lu G., Meier K.E., Jaffa A.A., Rosenzweing S.A., Egan B.M. (1998). Oleic acid and angiotensin II induce a synergistic mitogenic response in vascular smooth muscle cells. Hypertension.

[23] Rao G.N., Alexander R.W., Runge M.S. (1995). Linoleic acid and its metabolites, hydroperoxyoctadecadienoic acids, stimulate c-fos, c-jun and c-myc mRNA expression, MAP kinase activation and growth in rat aortic smooth muscle cells. J. Clin. Investig..

[24] Mu Y.M., Yanase T., Nishi Y., Tanaka A., Saito M., Jin C.H., Mukasa C., Okabe T., Nomura M., Goto K. (2001). Saturated FFAs, palmitic acid and stearic acid, induce apoptosis in human granulosa cells. Endocrinology.

[25] Eitel K., Staiger H., Brendel M.D., Brandhorst D., Bretzel R.G., Haring H.U., Kellerer M. (2002). Different role of saturated and unsaturated fatty acids in beta-cell apoptosis. Biochem. Biophys. Res. Commun..

[26] Akazawa Y., Cazanave S., Mott J.L., Elmi N., Bronk S.F., Kohno S., Charlton M.R., Gores G.J. (2010). Palmitoleate attenuates palmitate induced Bim and PUMA up-regulation and hepatocyte lipoapoptosis. J. Hepatol..

[27] Suzuki J., Akahane K., Nakamura J., Naruse K., Kamiya H., Himeno T., Nakamura N., Shibata T., Kondo M., Nagasaki H. (2011). Palmitate induces apoptosis in Schwann cells via both ceramide-dependent and independent pathways. Neuroscience.

[28] Pennisi E.M., Garibaldi M., Antonini G. (2018). Lipid myopathies. J. Clin. Med..

[29] Frago L.M., Canelles S., Freire-Regatillo A., Argente-Arizón P., Barrios V., Argente J., Garcia-Segura L.M., Chowen J.A. (2017). Estradiol uses different mechanisms in astrocytes from the hippocampus of male and female rats to protect against damage induced by palmitic acid. Front. Mol. Neurosci..

[30] Park J., Lee J., Song K.D., Kim S.J., Kim D.C., Lee S.C., Son Y.J., Choi H.W., Shim K.S. (2021). Growth factors improve the proliferation of Jeju black pig muscle cells by regulating myogenic differentiation and growth-related genes. Anim. Biosci..

[31] Lee J., Park J., Choe H., Shim K.S. (2022). Insect peptide CopA3 promotes proliferation and PAX7 and MYOD expression in porcine muscle satellite cells. J. Anim. Sci. Technol..

[32] Wang R., Wang W., Ao L., Wang Z., Hao X., Zhang H. (2017). Benzo[a]pyrene-7,8-diol-9,10-epoxide suppresses the migration and invasion of human extravillous trophoblast HTR-8/SVneo cells by downregulating MMP2 through inhibition of FAK/SRC/PI3K/AKT pathway. Toxicology.

[33] Song W., Liu P., Li H., Ding S. (2022). Large-Scale Expansion of Porcine Adipose-Derived Stem Cells Based on Microcarriers System for Cultured Meat Production. Foods.

[34] Park J., Lee J., Shim K.S. (2023). Effects of heat stress exposure on porcine muscle satellite cells. J. Therm. Biol..

[35] Baik M., Nguyen T.H., Jeong J.Y., Piao M.Y., Kang H.J. (2015). Effects of Castration on Expression of Lipid Metabolism Genes in the Liver of Korean Cattle. Asian-Australas. J. Anim. Sci..

[36] Livak K.J., Schmittgen T.D. (2001). Analysis of relative gene expression data using real time quantitative PCR and the 2^−ΔΔCt^ method. Methods.

[37] Chawla A., Repa J.J., Evans R.M., Mangelsdorf D.J. (2001). Nuclear receptors and lipid physiology: Opening the X-files. Science.

[38] Maurin A.C., Chavassieux P.M., Vericel E., Meunier P.J. (2002). Role of polyunsaturated fatty acids in the inhibitory effect of human adipocytes on osteoblastic proliferation. Bone.

[39] van den Heuvel S. (2005). Cell cycle regulation. Wormbook.

[40] Yonezawa T., Haga S., Kobayashi Y., Katoh K., Obara Y. (2008). Unsaturated fatty acids promote proliferation via ERK1/2 and Akt pathway in bovine mammary epithelial cells. Biochem. Biophys. Res. Commun..

[41] Lu G., Morinelli T.A., Meier K.E., Rosenzweing S.A., Egan B.M. (1996). Oleic acid-induced mitogenic signaling in vascular smooth muscle cells. A role for protein kinase C. Circ. Res..

[42] Mattern H.M., Hardin C.D. (2007). Vascular metabolic dysfunction and lipotoxicity. Physiol. Res..

[43] Zeng X., Zhu M., Liu X., Chen X., Yuan Y., Li L., Liu J., Lu Y., Cheng J., Chen Y. (2020). Oleic acid ameliorates palmitic acid induced hepatocellular lipotoxicity by inhibition of ER stress and pyroptosis. Nutr. Metab..

[44] Peng J., Yang M., Li G., Zhang X., Huang Y., Tang Y. (2021). Effects of palmitic acid and eicosapentaenoic acid on angiogenesis of porcine vascular endothelial cells. Vet. Med. Sci..

[45] Grabiec K., Gajewska M., Milewska M., Błaszczyk M., Grzelkowska-Kowalczyk K. (2014). The influence of high glucose and high insulin on mechanisms controlling cell cycle progression and arrest in mouse C2C12 myoblasts: The comparison with IGF-I effect. J. Endocrinol. Investig..

[46] Zhang G., Chen X., Lin L., Wen C., Rao S. (2012). Effects of fatty acids on proliferation and differentiation of myoblast. Wei Sheng Yan Jiu = J. Hyg. Res..

[47] Cossu G., Borello U. (1999). Wnt signaling and the activation of myogenesis in mammals. EMBO J..

[48] Weintraub H., Davis R., Tapscott S., Thayer M., Krause M., Benezra R., Blackwell T.K., Turner D., Rupp R., Hollenberg S. (1991). The myoD gene family: Nodal point during specification of the muscle cell lineage. Science.

[49] Sun B., Sun J., Li Q., Wang Y., Wang E., Jin H., Hua H., Jin Q., Li X. (2023). Study on the effect of oleic acid-induced lipogenic differentiation of skeletal muscle satellite cells in Yanbian cattle and related mechanisms. Animals.

[50] Zhao D.D., Liu C.C., Jia M.Y., Yang Y., Ye F., Yan Y.Q. (2012). Progress in the study of upstream transcriptional regulatory elements of muscle-specific gene promoters. Chin. J. Cell Biol..

[51] Hasty K.A., Wu H., Byrne M., Goldring M.B., Seyer J.M., Jaenisch R., Krane S.M., Mainardi C.L. (1993). Susceptibility of type I collagen containing mutated alpha 1(1) chains to cleavage by human neutrophil collagenase. Matrix.

[52] Apponi L.H., Corbett A.H., Pavlath G.K. (2011). RNA-binding proteins and gene regulation in myogenesis. Trends Pharmacol. Sci..

[53] Li X.Z., Yan Y., Zhang J.F., Sun J.F., Sun B., Yan C.G., Choi S.H., Johnson B.J., Kim J.K., Smith S.B. (2019). Oleic acid in the absence of a PPARγ agonist increases adipogenic gene expression in bovine muscle satellite cells. J. Anim. Sci..

[54] Stewart C.E., Rittweger J. (2006). Adaptive processes in skeletal muscle: Molecular regulators and genetic influences. J. Musculoskelet. Neuronal Interact..

[55] Fujimoto Y., Onoduka J., Homma K.J., Yamaguchi S., Mori M., Higashi Y., Makita M., Kinoshita T., Noda J.I., Itabe H. (2006). Long-chain fatty acids induce lipid droplet formation in a cultured human hepatocyte in a manner dependent of Acyl-CoA synthetase. Biol. Pharm. Bull..

[56] Kohjima M., Enjoji M., Higuchi N., Kato M., Kotoh K., Nakashima M., Nakamuta M. (2009). The effects of unsaturated fatty acids on lipid metabolism in HepG2 cells. In Vitro Cell. Dev. Biol. -Anim..

[57] Belal S.A., Kang D.R., Sivakumar A.S., Choe H.S., Shim K.S. (2019). Effect of long chain fatty acids on triacylglycerol accumulation, fatty acid composition and related gene expression in primary cultured bovine satellite cells. Anim. Biotechnol..

[58] Zhang J., Li Q., Nogoy K.M., Sun J., Sun B., Wang Y., Tang L., Yu J., Jin X., Li X. (2021). Effect of palmitoleic acid on the differentiation of bovine skeletal muscle satellite cells. J. Anim. Sci. Technol..

[59] Herzig S., Shaw R.J. (2018). AMPK: Guardian of metabolism and mitochondrial homeostasis. Nat. Rev. Mol. Cell Biol..

[60] Mersmann H.J., Ding S.T. (2001). Fatty acids modulate porcine adipocyte differentiation and transcripts for transcription factors and adipocyte-characteristic proteins. J. Nutr. Biochem..

[61] Sanosaka M., Minashima T., Suzuki K., Watanabe K., Ohwada S., Hagino A., Rose M.T., Yamaguchi T., Aso H. (2008). A combination of octanoate and oleate promotes in vitro differentiation of porcine intramuscular adipocytes. Comp. Biochem. Physiol. Part B Biochem. Mol. Biol..

