# Supplementary material for: The Effects of Oleic Acid and Palmitic Acid on Porcine Muscle Satellite Cells
Source: Foods. 2024 Jul 12;13(14):2200. doi: 10.3390/foods13142200 (PMC11276066; doi:10.3390/foods13142200)
Supplement: Supplementary file 1 [file foods-13-02200-s001.zip › foods-3052034-supplementary.pdf]

Table: S1: Summary of primers of target reference genes used for qPCR analysis

Gene	Sequence	References
GAPDH	F: 5′-ACCCAGAAGACTGTGGATGG-3′<br>R: 5′-AAGCAGGGATGATGTTCTGG-3′	Park et al., [34]
PAX7	F: 5′-TCCAGCTACTCCGACAGCTT-3′<br>R: 5′-TGCTCAGAATGCTCATCACC -3′	
MYOD	F: 5′-GTGCAAACGCAAGACCACTA -3′<br>R: 5′-GCTGATTCGGGTTGCTAGAC-3′	Lee et al., [31]
MYOG	F: 5′-CCACTTCTATGACGGGGAAA -3′<br>R: 5′-GGTCCACAGACACGGACTTC-3′	
PPARγ	F: 5′-TGGCCATTCGCATCTTTCAG-3′<br>R: 5′-ATCTCGTGGACGCCATACTT-3′	Song et al., [33]
FABP4	F: 5′-AGAAGTGGGAGTGGGCTTTG-3′<br>R: 5′-ATGATCAGGTTGGGTTTGGC-3′	
C/EBPα	F: 5′-GAGCCCGGCAACTCTAGTAT-3′<br>R: 5′-CCCTACTCGGTAGGAATCGG-3′	
Plin1	F: 5′-CAGTTCACAGCTGCCAATGA-3′<br>R: 5′-TTCAGCTCAGAGGCGATCTT-3′	
FAS	F: 5′-ATCGAGTGCATCAGGCAAGT-3′<br>R: 5′-TGTGAGCACATCTCGAAAGCCA-3′	Baik et al. [35]
LPL	F: 5′-CTTGCCACCTCATTCCTG-3′<br>R: 5′-ACCCAACTCTCATACATTCCTG-3′	

References

31. Lee, J.; Park, J.; Choe, H.; Shim, K.S. Insect peptide CopA3 promotes proliferation and PAX7 and MYOD expression in porcine muscle satellite cells. J Anim Sci Technol. **2022**, 64(6), 1132-1143.
33. Song, W.; Liu, P.; Li, H.; Ding, S. Large-Scale Expansion of Porcine Adipose-Derived Stem Cells Based on Microcarriers System for Cultured Meat Production. Foods. **2022**, 11, 3364.
34. Park, J.; Lee, J.; Shim, K.S. 2023. Effects of heat stress exposure on porcine muscle satellite cells. Journal of Thermal Biol. **2023**, 114, 103569.
35. Baik, M.; Nguyen, T.H.; Jeong, J.Y.; Piao, M.Y.; Kang, H.J. Effects of Castration on Expression of Lipid Metabolism Genes in the Liver of Korean Cattle. Asian-Australasian J. Anim. Sci. **2015**, 28(1), 127-134.
